# Resilient Aspirants: Women's Candidacies and Election in Times of COVID-19

**DOI:** 10.1017/S1743923X20000537

**Published:** 2020-07-30

**Authors:** Malu A. C. Gatto, Debora Thome

**Affiliations:** 1 University College London; 2 Universidade Federal Fluminense

**Keywords:** COVID-19, women's representation, elections, candidate recruitment, Brazil

## Abstract

The COVID-19 pandemic has gendered implications for women's time and resources. The use of informal institutions that pose obstacles to women's electoral viability may also be particularly consequential at a time of rapid change, when election dates and procedures are being amended because of health concerns. Together, these dynamics suggest that the COVID-19 pandemic may impact women's electoral participation, support, and viability in meaningful ways. The November 2020 Brazilian municipal elections provide an opportunity to explore this. Employing data from an original survey of eligible individuals and aspirant candidates, we find that the main obstacle to women's representation is not personal political ambition or efforts but women's perceptions of their access to support for their candidacies. In the face of greater challenges, resilient aspirants are choosing to work harder to compensate for potential losses in campaign support and funds.

Less access to financial resources, less time availability, and more restricted social networks are common barriers to women's electoral prospects (Inglehart and Norris 2003). Additionally, parties’ reliance on unwritten yet persistent practices and norms (i.e., informal institutions) of candidate recruitment and campaign support allocation can serve as obstacles to women's election to office, sometimes even when formal institutions favor women (Bjarnegård and Zetterberg 2019).

As a number of emerging analyses show, the COVID-19 pandemic seems to be decreasing women's financial stability, increasing their shares of unpaid domestic and care work, and restricting their possibilities to establish key contacts (Wenham, Smith, and Morgan 2020). Times of change—such as that prompted by the pandemic—also increase opportunities for informal practices to flourish (Waylen 2014). Together, these dynamics suggest that the COVID-19 pandemic may be detrimental to women's electoral prospects.

In particular, we anticipate that changes in electoral dynamics may impact political aspirations, perceptions of electoral viability, and expectations of access to resources in gendered ways. More specifically, by affecting individuals’ household responsibilities, financial resources, and emotional stability, the pandemic could impose higher personal costs of running for office, *demotivating women's candidacies*. In addition, constraints on face-to-face interactions and the reduction of campaign time could increase traditional forms of political capital and incumbents’ advantages (Pereira and Rennó 2001), *making women less certain of their electoral prospects*. Finally, disruptions to interactions with party brokers could increase candidates’ reliance on resources attained through informal institutions and established networks, *decreasing women's perceptions of their access to valuable campaign resources*. The 2020 Brazilian elections provide an opportunity to explore these possible scenarios.

As a result of the pandemic, Brazil delayed to November 2020 municipal elections for mayor and city councilors that had originally been scheduled for October. Voting in Brazil is mandatory and done only in person. Changes to campaign procedures, formal isolation protocols, and individuals’ fears of personal interactions are likely to drastically change electoral dynamics; personal interactions between party leaders and candidates and between candidates and voters have traditionally been crucial in Brazil, particularly at the local level (Barreira 2006).

In spite of the adoption of a gender quota for municipal elections in 1995, the persistence of gendered political practices and employment of informal institutions in Brazil have largely kept women out of elected office (Wylie and dos Santos 2016). Since 2018, women occupy 15% of the lower house of Congress, up from 10% in 2014.

This 50% increase in women's representation has not gone unnoticed. Some authors associate it with the newly instituted reservation of 30% of public campaign finance for women (Haje 2019). Meanwhile, others have observed that, as in the United States, the electoral popularity (and subsequent election) of a far-right and misogynist candidate, Jair Bolsonaro, might have increased women's costs of not running (Dittmar 2020). Within this context, and considering that many politicians start their careers at the local level, the 2020 elections were expected to be a turning point for women's political participation in Brazil.

The pandemic, however, could change this scenario. Employing data from an original survey of party members (including individuals eligible for candidacy and active aspirants to elected office), this research note provides an exploratory investigation of how COVID-19 may impact women's electoral opportunities. We find that COVID-19 did not disproportionately impact women's plans to run or their evaluations of their electoral chances, but it decreased women's perceptions of their levels of access to campaign support and resources. These findings reinforce previous work that shows women to be particularly *resilient aspirants* even amid unexpected adversity.

## DATA AND METHODS

COVID-19 is likely to impact candidacy decisions and campaign strategies heterogeneously, depending on individuals’ backgrounds and levels of political experience. To attain a diverse sample of respondents, we partnered with the Paraná state branch of the Partido Republicano da Ordem Social (Social Order Republican Party or PROS). The party branch was responsible for disseminating the survey link to all their registered members in Paraná.

Created in 2013, PROS is one of the newest parties in Brazil's highly fragmented system of 33 registered parties and offers a good representation of a typical Brazilian party. As Appendix A in the supplementary materials shows, PROS is the party closest to the mean value of Brazilian parties’ left-right ideological placement. Additionally, the overwhelming majority of politicians in Brazil belong to nonprogrammatic parties such as PROS, which offer particular career incentives (Zucco and Power 2019). Findings derived from a sample of respondents from the PROS should thus be generalizable to the majority of Brazilian parties.

We collected a sample of 139 responses through the online platform Qualtrics from June 4 to 21, 2020. Our recruitment strategy oversampled highly educated and politically active members: 66% of respondents had (and continue to have) candidacy plans (see Appendix B). To understand whether and how COVID-19 is likely to have gendered implications for women's political representation, we use answers to the survey questions to derive three types of dependent variables (see Table 1).

**Table 1.** Dependent variables

*Variable*	*Question/Answers*	*Value*
Candidacy plans	**Has the pandemic changed your 2020 candidacy plans?**	
	*Answer options*	
	Before the pandemic, I was planning to run, but now I will not.	–1
	I have never planned to run, and I still do not have any plans.	0
	I have always planned to run, and I still have these plans.	1
	Before the pandemic, I did not plan to run, but now I will.	2
Electoral chances	**How will COVID-19 impact your/your party's electoral chances?**	
	*Answer options*	
	My/my party's electoral chances will be lower.	–1
	My/my party's electoral chances will not change.	0
	My/my party's electoral chances will increase.	1
Resource access	How will COVID-19 impact access to campaign resource X?*	
	*Answer options*	
	Access to X resource will decrease.	–1
	Access to X resource will not change.	0
	Access to X resource will increase.	1

* Consists of six dependent variables: party funds, party leadership support, donations in money, donations in work, street campaigns, and social media campaigns.

Given our focus on the potential gendered impacts of COVID-19, our main independent variable is respondents’ gender. In our binary variable, *woman*, women respondents are assigned a value of 1. In our sample, 56 respondents (40%) are women.

We also add controls for respondents’ *age*, race (with the binary variable *white*), and household *income*. Since electoral campaigns require candidates’ contributions of money and time, we also control for whether they are the main *breadwinner* and whether *minors* live with them. We employ the variable *personal changes* to account for whether COVID-19 impacted respondents’ domestic and care responsibilities, finances, health, or emotional stability. Since individuals’ political opportunities can be shaped by their relationship to their parties and political experiences, we control for their party *membership length* (in years), number of *past candidacies*, and number of *past electoral successes*. Finally, given that our sample includes eligible individuals and aspirants, we control for whether a respondent plans to run for office in 2020 with the variable *aspirant*.^1^ Appendix C outlines our survey questionnaire, and Appendix D shows descriptive statistics for our variables.

## DISCUSSION

To simplify interpretation, we run ordinary least squares (OLS) models for all of our dependent variables; we also report results from ordered logit models in the Appendices G–I. Interestingly, the pandemic led 12 respondents (8.6%) to change their candidacy plans: five of them (two women) will no longer run and seven (three women) became encouraged to run. As shown in Appendix G, gender is not a statistically significant characteristic shaping candidacy decisions. Women also do not statistically differ from men in respect to their evaluations of how the pandemic will impact their or their parties’ electoral chances. As one woman explained, “I believe there are always adaptations to the new.”

**Figure 1.**
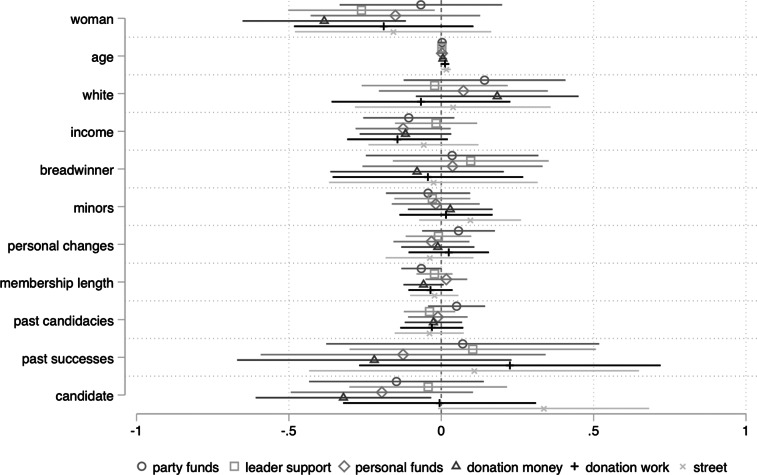
COVID-19's impact on campaign support and resources, OLS. *N* = 139.

However, women differ significantly from men in their evaluations of how COVID-19 will impact *access* to campaign support and resources (findings that hold when we restrict our models to aspirants only, as shown in Appendix H). Specifically, as shown in Figure 1, women are more likely than men to believe that COVID-19 will diminish donations in money and support from party brokers^2^—resources that are crucial for electoral success in an open-list proportional representation system (Jalalzai and dos Santos 2015).^3^

Since 2018, 30% of public campaign funds distributed to parties have been reserved for women. Yet party brokers have discretion over the distribution of these funds. In the 2018 elections, parties largely used this reserved 30% to fund the campaigns of women incumbents and those of women running mates of male primary candidates.

Even in this context, our findings suggest that women are as confident as men about their access to public campaign funds. However, the unequal distribution of these public resources and the high costs associated with campaigns in a candidate-centric system ultimately mean that these additional sources of finance remain important for candidates’ electoral success. Women's perception that the pandemic will decrease their access to monetary donations is thus a relevant finding in the run-up to the 2020 elections. Among eligible women, perceptions of lower access to funds could also impact decisions to run in the future.

Additionally, PROS-Paraná's efforts to promote women's leadership through daily online meetings do not seem to have been sufficient to make women as confident as men about support from party leaders. This is a significant finding, particularly given our oversampling of politically active party members.

Answers to open-ended questions indicate that to compensate for potential losses in support and funds, women are increasing their political engagement. Remarkably, some women do not seem to view recent changes as a handicap but as an opportunity. As one respondent conveyed, “My performance has increased as I now have to try to do more and better.” Crucially, although some men mentioned problems concerning money and party dynamics, women's answers most commonly focused on adaptations to their campaign strategies. As another woman said, “We have been more participative in social activities, as this strengthens our relationship with others.”

In sum, our results indicate that women are particularly *resilient aspirants*: even amid a crisis that has gendered implications for personal time and resources, the main obstacle to women's prospects is not personal political ambition or efforts but women's perceptions of their access to campaign resources and party brokers’ support. These findings are aligned with existing scholarship that shows that women's underrepresentation in politics cannot be explained by their personal attributes or constraints, or by their lower levels of political ambition and perceptions of electoral viability than men, but by the different levels of campaign support they encounter (Piscopo and Kenny 2020): a pattern they seem to be aware of and that is reproduced during crises.

## Footnotes

1. Not included in models of *candidacy plans*.

2. Our measure of personal changes due to *COVID-19* is not statistically significant in any of our models, suggesting that within our sample, personal adversities brought up by the pandemic have not influenced respondents’ decision to run or their assessments of their electoral viability (see Appendices G–I). In logistic regressions using the individual components of *personal changes* as dependent variables, we additionally find that gender is not a statistically significant explanatory variable (Appendix F). A note of caution is in order when interpreting these results: our sample is composed of highly educated respondents whose income might not have been as threatened by the pandemic.

3. This is not due to gendered differences in assessments of the importance of these resources, as shown in Appendix I.

## Supplementary material

For supplementary material accompanying this paper visit http://dx.doi.org/10.1017/S1743923X20000537. click here to view supplementary material
