# Supplementary material for: Resilient Aspirants: Women's Candidacies and Election in Times of COVID-19
Source: Politics & Gender. 2020 Jul 30:1–8. doi: 10.1017/S1743923X20000537 (PMC7562784; doi:10.1017/S1743923X20000537)
Supplement: Supplementary file 1 [file S1743923X20000537sup001.docx]

**Resilient aspirants: Women’s candidacies and election in times of Covid-19**

Malu A. C. Gatto^[[1]](#footnote-1)^* and Debora Thome^[[2]](#footnote-2)^+

**Appendix**

**Appendix A. Case selection**

**Appendix B. Sample characteristics**

**Appendix C. Survey questions and variables**

**Appendix D. Descriptive statistics**

**Appendix E. Frequency distribution of dependent variables**

**Appendix F. Results, personal changes due to Covid-19**

**Appendix G. Results, candidacy plans and perceived viability**

**Appendix H. Results, expected impact of Covid-19 on campaign support and resources**

**Appendix I. Results, importance of campaign support and resources**

**Appendix A. Case selection**

**Figure 1. Brazilian parties’ placement on the left-right ideology scale (2018)**

Note: Figure produced using data from Rodrigues and Power (2019), which places all 35 Brazilian parties on a left-right ideology scale—where negative values correspond to left-leaning ideology, 0 to the center, and positive values indicate right-leaning ideology. The vertical line takes the mean value of left-right ideology (0.095) to illustrate each party’s distance from the mean. To aid the visualization of variation across left-right ideology (x-axis), we plot it against parties’ identification numbers (i.e., variation on the y-axis does not have a meaningful interpretation). All figures were compiled with *plotplain* (Bischof 2017).

**Appendix B. Sample characteristics**

|  | **Population** | **% known pop.** | **Sample** | **% sample** | **Difference** |
| --- | --- | --- | --- | --- | --- |
| Women | 4238 | 0.352 | 56 | 0.403 | 0.051 |
| Men | 7817 | 0.648 | 83 | 0.597 | -0.051 |
| <20 | 117 | 0.010 | 1 | 0.007 | -0.003 |
| 21 to 25 | 775 | 0.064 | 5 | 0.036 | -0.028 |
| 26 to 30 | 1114 | 0.092 | 9 | 0.065 | -0.028 |
| 31 to 35 | 1315 | 0.109 | 17 | 0.122 | 0.013 |
| 36 to 40 | 1503 | 0.125 | 22 | 0.158 | 0.034 |
| 41 to 45 | 1589 | 0.132 | 26 | 0.187 | 0.055 |
| 46 to 50 | 1568 | 0.130 | 26 | 0.187 | 0.057 |
| 51 to 55 | 1445 | 0.120 | 19 | 0.137 | 0.017 |
| 56 to 60 | 1093 | 0.091 | 8 | 0.058 | -0.033 |
| 61 to 65 | 709 | 0.059 | 5 | 0.036 | -0.023 |
| >65 | 818 | 0.068 | 1 | 0.007 | -0.061 |
| Primary (incomplete) | 958 | 0.086 | 1 | 0.007 | -0.079 |
| Primary (complete) | 1840 | 0.165 | 5 | 0.036 | -0.129 |
| Secondary (incomplete) | 834 | 0.075 | 8 | 0.058 | -0.017 |
| Secondary (complete) | 4682 | 0.419 | 21 | 0.151 | -0.268 |
| Tertiary (incomplete) | 711 | 0.064 | 21 | 0.151 | 0.087 |
| Tertiary (complete) | 1794 | 0.160 | 33 | 0.237 | 0.077 |
| Postgraduate | 360 | 0.032 | 50 | 0.360 | 0.328 |

Note: Population corresponds to registered members of the PROS in the state of Paraná. Information on population characteristics were provided by the party. The total population size is 13,358. However, the official party registry did not contain information on gender for 1,303 members, age for 1,312 members, and education for 2,161 members. Population shares are calculated from the “known populations,” which refer to the number of people for whom the party has data for each type of information. The sample size is 139. Differences between population and sample characteristics were calculated by subtracting a category’s share in the population from its share in the sample. Positive numbers can thus be interpreted as oversampling (and negative numbers as under-sampling). Our sample is reasonably similar to the population on gender and age, but significantly more highly educated. This is likely the result of the type of party members we managed to recruit: those who are more actively engaged with the party: 66% of respondents had (and continue to have) plans to run for elected office in 2020.

**Appendix C. Survey questions and variables**

*woman*

0 = Man

1 = Woman

*age*

17-67

*white*

0 = All other

1 = White

*Income* (household)

1 = Less than two minimum wages

2 = Two to four minimum wages

3 = Five to ten minimum wages

4= More than ten minimum wages

*breadwinner* (Are you the breadwinner?)

0 = No

1 = Yes

*minors* (total number of minors who live with respondent calculated based on answers to the questions: Do you have kids?; What is their age?; Do they live with you?)

1-4

*care* (Did the pandemic increase the time you spend taking care of children and elderly and/or doing housework?)

0 = No

1 = Yes

*Job* (Did you or anyone who lives with you lose a job?)

0 = No

1 = Yes

*illness* (Did you or anyone who lives with you get infected with Covid-19?)

0 = No

1 = Yes

*death* (Did any of your relatives or friends die from Covid-19?)

0 = No

1 = Yes

*income* (Did you or anyone who lives with you and contributes to household finances, lose their income?)

0 = No

1 = Yes

*personal changes* (sum of *care*, *job*, *illness*, *death*, and *income*)

1-5

*membership length* (When did you get affiliated to the PROS?)

- 1. (years)

*past candidacies* (Have you ever been a candidate?)

0-9 (times)

*past successes* (Have you ever been elected?)

0 = No

1 = Yes

***candidacy plans*** (Has the pandemic changed your 2020 candidacy plans?)

-1 = Before the pandemic, I was planning to run, but now I will not.

0 = I have never planned to run and I still do not have any plans.

1 = I have always planned to run and I still have these plans.

2 = Before the pandemic, I did not plan to tun, but now I will.

***electoral chances*** (How will Covid-19 impact yours/your party’s electoral chances?)

-1 = Will be lower

0 = Will not change

1 = Will increase

***resource importance*** battery (How would you describe the relevance of [X] for your electoral success/the electoral success of those you support?)

*importance: party funds*

0 = Not important

1 = Somewhat important

2 = Very important

*importance: support from party leaders*

0 = Not important

1 = Somewhat important

2 = Very important

*importance: personal funds to finance campaign*

0 = Not important

1 = Somewhat important

2 = Very important

*importance: private donations in money*

0 = Not important

1 = Somewhat important

2 = Very important

*importance: private donations in hours of work*

0 = Not important

1 = Somewhat important

2 = Very important

*importance: street campaign*

0 = Not important

1 = Somewhat important

2 = Very important

***resource access*** battery (How do you think Covid-19 will impact access to [X]?)

*access: party funds*

-1 = Will decrease

0 = Will not change

1 = Will increase

*access: support from party leaders*

-1 = Will decrease

0 = Will not change

1 = Will increase

*access: personal funds to finance campaign*

-1 = Will decrease

0 = Will not change

1 = Will increase

*access: private donations in money*

-1 = Will decrease

0 = Will not change

1 = Will increase

*access: private contributions in hours of work*

-1 = Will decrease

0 = Will not change

1 = Will increase

*access: street campaign*

-1 = Will decrease

0 = Will not change

1 = Will increase

***open-ended question*** (Has Covid-19 changed your political engagement? If so, how?)

**Appendix D. Descriptive statistics**

| **Dependent Variable** | **N** | **Mean** | **Std. Dev.** | **Min** | **Max** |
| --- | --- | --- | --- | --- | --- |
| *candidacy plans* | 139 | 0.727 | 0.612 | -1 | 2 |
| *electoral chances* | 139 | -0.014 | 0.712 | -1 | 1 |
| *party funds (change)* | 139 | -0.122 | 0.717 | -1 | 1 |
| *leader support (change)* | 139 | 0.345 | 0.645 | -1 | 1 |
| *personal funds (change)* | 139 | -0.086 | 0.737 | -1 | 1 |
| *donation money (change)* | 139 | -0.230 | 0.745 | -1 | 1 |
| *donation work (change)* | 139 | 0.101 | 0.792 | -1 | 1 |
| *street campaign (change)* | 139 | -0.158 | 0.870 | -1 | 1 |
| **Independent Variable** |  |  |  |  |  |
| *woman* | 139 | 0.403 | 0.492 | 0 | 1 |
| *age* | 139 | 43.295 | 10.083 | 17 | 67 |
| *white* | 139 | 0.647 | 0.479 | 0 | 1 |
| *income* | 139 | 2.338 | 0.873 | 1 | 4 |
| *breadwinner* | 139 | 0.640 | 0.482 | 0 | 1 |
| *minors* | 139 | 0.856 | 0.937 | 0 | 4 |
| *personal changes* | 139 | 1.496 | 1.052 | 0 | 4 |
| *membership length* | 139 | 1.252 | 1.930 | 0 | 10 |
| *past candidacies* | 139 | 0.576 | 1.367 | 0 | 9 |
| *past successes* | 139 | 0.094 | 0.292 | 0 | 1 |
| *candidate* | 139 | 0 .712 | 0 .454 | 0 | 1 |

**Appendix E. Frequency distribution of dependent variables**

**Figure 2. Frequency distribution of *candidacy plans* and *electoral chances***

**Figure 3. Frequency distribution of *resource access***

**Appendix F. Results, personal changes due to Covid-19**

**Figure 4. Changes in personal life due to Covid-19, Logit**

Note: N=139. One component of *personal changes*, *illness*, is not included in the analysis as no woman surveyed reported having been infected by the virus (i.e., gender perfectly explains the outcome).

**Appendix G. Results, candidacy plans and perceived viability**

**Figure 5. Candidacy plans and perceived electoral viability, OLS**

Note: N=139.

**Figure 6. Candidacy plans and perceived electoral viability, Ordered logit**

Note: N=139.

**Appendix H. Results, expected impact of Covid-19 on campaign support and resources**

**Figure 7. Covid-19’s impact on campaign support and resources among aspirants only, OLS**

Note: N=99.

**Figure 8. Covid-19’s impact on campaign support and resources, Ordered logit**

Note: N=139.

**Appendix I. Results, importance of campaign support and resources**

**Figure 9. Importance of campaign support and resources, OLS**

Note: N=139.

**Figure 10. Importance of campaign support and resources, Ordered logit**

Note: N=139. Model of *leader support* does not include control for *past successes* as this variable perfectly explains the outcome.

**References**

Bischof, Daniel. 2017. “New graphic schemes for stata: Plotplain and plottig.” *Stata Journal*, *17*(3): 748–759.

Power, Timothy J., and Rodrigo Rodrigues-Silveira. 2019. “Mapping Ideological Preferences in Brazilian Elections, 1994-2018: A Municipal-Level Study.” *Brazilian Political Science Review*, *13*(1). *First View*.

1. * University College London (UCL), [m.gatto@ucl.ac.uk](mailto:m.gatto@ucl.ac.uk) [↑](#footnote-ref-1)
2. + Universidade Federal Fluminense (UFF), [deborathome@gmail.com](mailto:deborathome@gmail.com) [↑](#footnote-ref-2)
